# Supplementary material for: Effects of electric vehicle charging stations on the economic vitality of local businesses
Source: Nat Commun. 2024 Sep 4;15:7437. doi: 10.1038/s41467-024-51554-9 (PMC11375121; doi:10.1038/s41467-024-51554-9)
Supplement: Supplementary file 1 — Supplementary Information [file 41467_2024_51554_MOESM1_ESM.pdf]

Supplementary Information: Effects of Electric Vehicle Charging Stations on the Economic Vitality of Local Businesses

Yunhan Zheng[1,2,*], David R. Keith[3], Shenhao Wang[4], Mi Diao[5], Jinhua Zhao[6]

1 Department of Civil and Environmental Engineering, Massachusetts Institute of Technology, USA

2 Singapore–MIT Alliance for Research and Technology Centre (SMART), Singapore

3 Centre for Sustainability and Business, Melbourne Business School, Australia

4 Department of Urban and Regional Planning, University of Florida, USA

5 College of Architecture and Urban Planning, Tongji University, China

6 Department of Urban Studies and Planning, Massachusetts Institute of Technology, USA

***** To whom correspondence should be addressed. E-mail: yunhan@mit.edu

Contents

S1 Literature review **S2**
S1.1 Modes of EV charging S2
S1.1.1 Level 1, level 2, and fast-charging S2
S1.1.2 Home, public, and workplace charging S2
S1.2 Characteristics of EV buyers S3
S1.3 EV charger usage patterns S3
S1.4 Business models of EV chargers S4
S1.5 Government incentives for promoting EV adoption S4

S2 Descriptive statistics **S5**

S3 Propensity score matching results **S10**

S4 Variation in treatment effects by distance **S12**

S5 Heterogeneity of Effects by POI and EV Charger Type **S15**

S6 Effects of EVCS on customer counts by different income groups **S18**

S7 Sensitivity analysis regarding disadvantaged community definition **S18**

S8 EVCS costs **S19**

S1 Literature review

Prior studies indicate that enhancing Electric Vehicle (EV) charging infrastructure can positively influence individuals' inclination to embrace EVs. Despite extensive investigations into behavioral dynamics, preferences, and policy frameworks surrounding EV adoption, literature concerning the existing EV charging market is relatively scarce. Here we provide a literature review focusing on the EV charging modalities, characteristics of EV buyers, EVCS usage patterns, business models of EVCS, and government incentives aimed at promoting EV adoption.

S1.1 Modes of EV charging

S1.1.1 Level 1, level 2, and fast-charging

EV chargers are categorized based on the rate at which they charge batteries, including Alternating Current (AC) Level 1, AC Level 2, and Direct Current (DC) fast charging.

- Level 1 chargers utilize a 120-volt (V) AC plug with a typical power output of 1 kW. It takes approximately 12 to 31 hours to charge an EV to fulfill a 100 km driving range [1].
- Level 2 chargers provide charging through either 240 V (typical in residential applications) or 208 V (typical in commercial applications) electrical service. These chargers typically have a power output ranging from 7 kW to 19 kW, taking between 3 to 6 hours to fulfill a 100 km driving range [1].
- DC fast chargers operate at 400 V to 1000 V DC, with a power output ranging from 50 kW to 350 kW. They require 20 to 80 minutes to fulfill a 100 km driving range [1].

S1.1.2 Home, public, and workplace charging

Most drivers of EVs in the United States charge their vehicles overnight at home using level 1 or level 2 chargers [2]. The capability of drivers to set up home charging hinges on whether they have access to a private off-street parking area, usually in the form of a driveway or garage. However, EV ownership is often concentrated in urban or densely populated areas, which may lead to a shortage of access to off-street parking and EV charging, which necessitates public charging.

General public charging uses Level 1, Level 2 or DC fast charging. While Level 2 charging stations are often situated in places where vehicle owners spend extended periods, such as shopping centers, airports, hotels, government offices, and various businesses, fast chargers are commonly located along highway routes [3, 4].

The third category encompasses workplace charging. Charging facilities at the workplace offer a significant opportunity for EV drivers, particularly those facing challenges in accessing home charging infrastructure. In scenarios where primary or sole reliance on workplace charging is possible, the provision of charging amenities by employers becomes especially impactful, particularly for fostering EV adoption among individuals lacking off-street parking facilities for home charging [3].

S1.2 Characteristics of EV buyers

Prior studies have indicated that in California, early adopters of EVs tend to be predominantly male, possess higher incomes, hold advanced levels of education, own homes, have multiple vehicles within their household, and have access to home charging facilities [5, 6, 7]. A study conducted using survey data from Plug-in Electric Vehicle (PEV) buyers in California between 2012 and 2017 revealed that the average household income of buyers is approximately \$187,000 per year. Additionally, 83% of buyers are homeowners, and 73% are male [5]. Another study, based on a cohort survey of PEV owners in California conducted in 2016 and 2017, revealed that over 80% of households owning PEVs have incomes exceeding the median household income in California [8]. However, research indicates that the profiles of PEV buyers are evolving over time and gradually diversifying beyond a limited group of high-income consumers. Lee et al. [5] finding that middle income buyers increased from <5% of PEV buyers in 2012 to 7.9% in 2017. discovered that middle-income buyers accounted for less than 5% of PEV buyers in 2012, but increased to 7.9% by 2017. The authors propose that this shift is a consequence of saturation among high-income PEV buyers and the diffusion of adoption to other income segments.

S1.3 EV charger usage patterns

The majority of charging events for PEVs occur at home. Findings from the Advanced PEV Travel and Charging Behavior Project (APEV project), which surveyed over 13,000 California PEV owners and lessees between 2015-2018, indicate that more than half of PEV owners exclusively charge at home, while 33% utilize a combination of home and other locations. Those who do not charge at home primarily rely on work charging, with some utilizing public charging opportunities [9, 8].

Although the utilization of public charging is less frequent compared to home and work charging, it serves as an important supplement to these primary locations. The APEV project reveals that approximately 3% of respondents rely solely on public charging, 13% utilize both home and public charging, 3% utilize both work and public charging, and 4% utilize all three types. In total, 23% of surveyed PEV owners make use of public charging to some extent [9, 8].

Regarding different types of chargers, the APEV data show that for battery electric vehicle (BEV) owners, Level 2 is the most frequently used charger at home and workplace while DC fast charger is used when charging in public locations. BEV owners who rely only on public charging locations mostly use DC fast chargers (about 1.5 days in a week) or Level 2 chargers (about 0.8 times per week). For plug-in hybrid electric vehicle (PHEV) owners, the DC fast chargers are unavailable, and more than 60% of PHEV owners used Level 1 chargers at home while mainly use Level 2 chargers at a workplace or public locations. PHEV owners who rely only on public charging locations use Level 2 chargers about 2.3 times per week [9, 8].

However, most prior research indicates that the utilization of public chargers in the United States remains low, often averaging fewer than one session per port per day [10, 11, 12]. A study conducted as part of the DOE-supported EV WATTS project revealed that as of March 2022, public EV chargers across the United States averaged 5.6 kWh per port per day (equivalent to 0.42 sessions

per port per day) for public Level 2 stations and 13.5 kWh per port per day (equivalent to 0.69 sessions per port per day) for public DC fast charging stations [12]. This poses a notable profitability challenge for the EV charger industry. Moreover, the temporal analysis reveals distinctive patterns: Level 2 station utilization peaks during weekdays, whereas DC fast chargers experience heightened utilization on weekends. Specifically, for Level 2 chargers, retail locations and leisure destinations exhibit peak utilization during weekdays from 10 a.m. to 2 p.m., extending into the evening. Conversely, for DC fast chargers, peak utilization occurs later in the day (2–6 p.m.) on both weekdays and weekends [12].

S1.4 Business models of EV chargers

Three business models exist concerning the relationship between EV charger companies and site hosts. The first, known as the "network-operator model" [13], involves companies developing and maintaining networks but selling hardware to host sites, which manage billing and access. Companies like ChargePoint and SemaConnect typically employ this model, focusing solely on installing and operating networks without retaining ownership. This setup allows host sites to establish payment structures and retain revenues, deducting fees paid to the operator [3]. The second model is the "owner-operator model" [13], where the EV charger company supplies and owns the hardware, manages billing and access, and collects revenues from the infrastructure. In such cases, the network operator may partner with a private host to install charging stations but retains control over pricing and assumes revenue risk. Companies like EVGo and Blink Network typically adopt this model [3]. The third model is the integrated model, exemplified by companies like Tesla, where EV charger companies are involved in all market functions, including manufacturing equipment, installing and managing networks, and collecting revenues. In this case, companies must recoup their charging infrastructure costs through user fees [3].

To offset the significant upfront expenses associated with installing hardware, especially for fast-charging stations, established industry players often utilize various payment structures. These typically consist of a combination of fixed fees, like annual or monthly subscriptions, usage-based fees where customers pay per kilowatt-hour consumed, time-based fees where customers pay for the duration of their charging session, and/or flat fees per charging session to access the charger [3].

Due to the relatively low utilization rate, covering the cost of EV chargers through usage fees, particularly for DC fast chargers, poses a significant challenge. Therefore, subsidies for EV charger deployment are crucial to address key barriers hindering their development.

S1.5 Government incentives for promoting EV adoption

As a frontrunner in EV adoption progress within the U.S., California benefits from a range of government supports for transitioning to electric vehicles. Between 2013 and 2022, the Legislature allocated a total of $3.5 billion to the California Air Resources Board for Low Carbon Transportation investments [14]. Nationally, initiatives like The Infrastructure Investment and Jobs Act (IIJA), passed in November 2021, earmark $7.5 billion for establishing a comprehensive charger network

nationwide [15]. At the regional level, efforts such as the California Electric Vehicle Infrastructure Project (CALeVIP), funded by the Alternative and Renewable Fuels and Vehicle Technology Program, encompass various regional EV charger incentive projects for public Level 2 and DC fast chargers. By March 2024, CALeVIP had invested $166.1 million in electric vehicle infrastructure projects, with $53.7 million in funding completed and $112.4 million reserved [16].

California has also made significant efforts to promote equity in EV adoption through legislative measures such as Senate Bill 535 (California Global Warming Solutions Act of 2006: Greenhouse Gas Reduction Fund) and Assembly Bill 1550 (Greenhouse gases: investment plan: disadvantaged communities). These bills mandate that a minimum of 25% of the Greenhouse Gas Reduction Fund be allocated to state programs aimed at reducing greenhouse gas emissions in disadvantaged communities [17, 18]. Additionally, there are EV charging station incentive programs that offer higher or exclusive rebates for low-income and disadvantaged communities. In 2018 and 2019, 69% of funding provided by CALeVIP was invested in Disadvantaged Communities or Low-Income Communities [16].

S2 Descriptive statistics

This section offers an overview of the data using descriptive statistics. Tables S1 and S2 provide descriptive statistics for the data in 2019 and the period from January 2021 to June 2023, respectively. Figure S1 illustrates the trends in EVCS counts by EVCS types, while Figure S2 highlights an anomalous surge in EV charging port counts attributed to a data management issue.

Table S1: Descriptive statistics for 2019 data

Variable	N	Mean	Std.dev	Min	Max
**Treated group**					
Monthly spending per POI (in $1000s)	68993	9.53	109.02	0.00	18915.94
Monthly number of customers per POI (in 1000s)	68993	0.13	0.26	0.00	5.84
Population density (people/km^2)	68993	3948.49	5051.27	0.00	43326.26
Building density (buildings/km^2)	68993	520.06	537.13	0.06	2583.57
Auto-oriented road miles per square mile	68993	2.66	3.18	0.00	27.08
Auto-oriented intersections per square mile	68993	8.16	12.11	0.00	105.60
Walkability Index score	68993	15.65	3.13	3.00	20.00
Median household income (in $1000s)	68993	81.42	38.96	9.19	250.00
Employed population percentage (%)	68993	66.14	9.94	5.61	86.54
Female percentage (%)	68993	49.43	5.46	25.73	61.66
Race: White population (%)	68993	59.01	20.69	3.15	95.73
Race: Black or African American population (%)	68993	6.32	7.11	0.00	77.20
Race: Native American population (%)	68993	0.57	1.00	0.00	15.07
Race: Asian population (%)	68993	18.45	18.19	0.00	93.77
Percentage of no-vehicle households (%)	68993	15.04	18.36	0.00	83.27
Monthly EV sales per 1000 people at the county level	68993	0.34	0.18	0.01	0.85
**Matched control group**					
Monthly spending per POI (in $1000s)	64656	14.06	63.65	0.00	2218.40
Monthly number of customers per POI (in 1000s)	64656	0.17	0.38	0.00	20.83
Population density (people/km^2)	64656	3338.20	3592.53	0.00	50191.27
Building density (buildings/km^2)	64656	546.65	555.09	0.02	2943.11
Auto-oriented road miles per square mile	64656	3.03	3.96	0.00	35.32
Auto-oriented intersections per square mile	64656	9.57	16.52	0.00	431.05
Walkability Index score	64656	15.53	2.83	3.17	20.00
Median household income (in $1000s)	64656	81.57	36.37	9.19	240.31
Employed population percentage (%)	64656	66.42	9.48	0.00	100.00
Female percentage (%)	64656	49.38	5.13	0.00	65.44
Race: White population (%)	64656	58.80	19.89	2.86	100.00
Race: Black or African American population (%)	64656	6.25	7.98	0.00	84.71
Race: Native American population (%)	64656	0.58	0.94	0.00	21.34
Race: Asian population (%)	64656	17.12	16.48	0.00	87.08
Percentage of no-vehicle households (%)	64656	10.19	12.95	0.00	87.76
Monthly EV sales per 1000 people at the county level	64656	0.34	0.20	0.01	0.85
**Unmatched control group**					
Monthly spending per POI (in $1000s)	1001994	6.86	35.12	-0.05	4360.30
Monthly number of customers per POI (in 1000s)	1001994	0.11	0.43	0.00	84.88
Population density (people/km^2)	1001994	2983.92	3396.08	0.00	59959.27
Building density (buildings/km^2)	1001994	567.90	582.53	0.00	3206.02
Auto-oriented road miles per square mile	1001994	2.27	3.35	0.00	39.53
Auto-oriented intersections per square mile	1001994	5.94	12.21	0.00	490.07
Walkability Index score	1001994	14.34	3.45	1.50	20.00
Median household income (in $1000s)	1001994	78.98	34.94	7.46	250.00
Employed population percentage (%)	1001994	64.83	8.88	0.00	100.00
Female percentage (%)	1001994	50.56	4.12	0.00	100.00
Race: White population (%)	1001994	62.05	19.80	0.00	100.00
Race: Black or African American population (%)	1001994	5.22	7.11	0.00	84.71
Race: Native American population (%)	1001994	0.80	2.13	0.00	100.00
Race: Asian population (%)	1001994	14.45	15.35	0.00	87.08
Percentage of no-vehicle households (%)	1001994	8.52	9.94	0.00	88.21
Monthly EV sales per 1000 people at the county level	1001994	0.31	0.19	0.01	0.85

Table S2: Descriptive statistics for 2021-2023 data

Variable	N	Mean	Std.dev	Min	Max
**Treated group**					
Monthly spending per POI (in $1000s)	657523	4.08	21.03	0.00	4211.39
Monthly number of customers per POI (in 1000s)	657523	0.06	0.36	0.00	63.38
Population density (people/km^2)	657523	2501.85	3496.10	0.00	71334.23
Building density (buildings/km^2)	657523	539.62	503.60	0.02	2924.75
Auto-oriented road miles per square mile	657523	2.67	3.55	0.00	39.53
Auto-oriented intersections per square mile	657523	7.91	13.20	0.00	142.44
Walkability Index score	657523	15.34	3.26	1.50	20.00
Median household income (in $1000s)	657523	87.10	34.88	12.90	250.00
Employed population percentage (%)	657523	65.31	8.72	0.87	100.00
Female percentage (%)	657523	50.29	4.35	0.00	100.00
Race: White population (%)	657523	56.69	17.69	4.63	100.00
Race: Black or African American population (%)	657523	5.35	6.29	0.00	78.28
Race: Native American population (%)	657523	0.84	1.58	0.00	34.90
Race: Asian population (%)	657523	16.30	14.70	0.00	90.98
Percentage of no-vehicle households (%)	657523	10.12	11.92	0.00	84.42
Monthly EV sales per 1000 people at the county level	657523	0.70	0.30	0.01	1.69
**Matched control group**					
Monthly spending per POI (in $1000s)	578296	6.27	42.87	0.00	14944.48
Monthly number of customers per POI (in 1000s)	578296	0.09	0.35	0.00	80.89
Population density (people/km^2)	578296	2378.49	2582.73	0.00	27380.54
Building density (buildings/km^2)	578296	613.24	608.26	0.02	3206.02
Auto-oriented road miles per square mile	578296	2.70	3.59	0.00	37.50
Auto-oriented intersections per square mile	578296	7.68	17.12	0.00	490.07
Walkability Index score	578296	15.19	2.76	2.00	20.00
Median household income (in $1000s)	578296	86.51	34.97	15.59	250.00
Employed population percentage (%)	578296	65.20	7.62	0.87	100.00
Female percentage (%)	578296	50.30	3.67	0.00	100.00
Race: White population (%)	578296	56.99	18.30	0.00	100.00
Race: Black or African American population (%)	578296	5.30	6.15	0.00	86.79
Race: Native American population (%)	578296	0.83	1.24	0.00	31.79
Race: Asian population (%)	578296	13.80	13.93	0.00	85.76
Percentage of no-vehicle households (%)	578296	7.61	7.71	0.00	74.08
Monthly EV sales per 1000 people at the county level	578296	0.58	0.29	0.01	1.69
**Unmatched control group**					
Monthly spending per POI (in $1000s)	1368684	2.89	32.96	0.00	18080.81
Monthly number of customers per POI (in 1000s)	1368684	0.04	0.91	0.00	597.63
Population density (people/km^2)	1368684	2354.73	2746.41	0.00	71334.23
Building density (buildings/km^2)	1368684	535.24	592.99	0.00	3206.02
Auto-oriented road miles per square mile	1368684	2.06	3.15	0.00	39.53
Auto-oriented intersections per square mile	1368684	4.91	10.01	0.00	490.07
Walkability Index score	1368684	13.57	3.62	1.00	20.00
Median household income (in $1000s)	1368684	84.01	35.13	8.67	250.00
Employed population percentage (%)	1368684	64.23	8.08	0.00	100.00
Female percentage (%)	1368684	50.23	3.87	0.00	100.00
Race: White population (%)	1368684	55.60	19.20	0.00	100.00
Race: Black or African American population (%)	1368684	5.23	6.71	0.00	86.79
Race: Native American population (%)	1368684	0.96	1.82	0.00	100.00
Race: Asian population (%)	1368684	14.04	14.81	0.00	100.00
Percentage of no-vehicle households (%)	1368684	7.22	7.12	0.00	94.95
Monthly EV sales per 1000 people at the county level	1368684	0.67	0.32	0.01	1.69

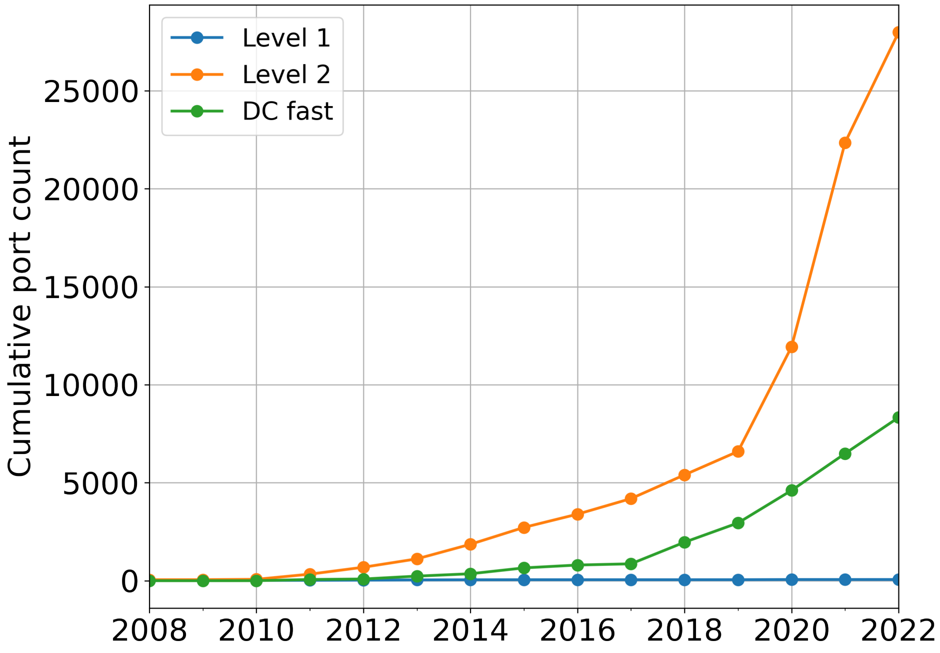

Figure S1: **Trends of EVCS counts by EVCS types.** Data source: the United States Department of Energy's Alternative Fuels Data Center [19].

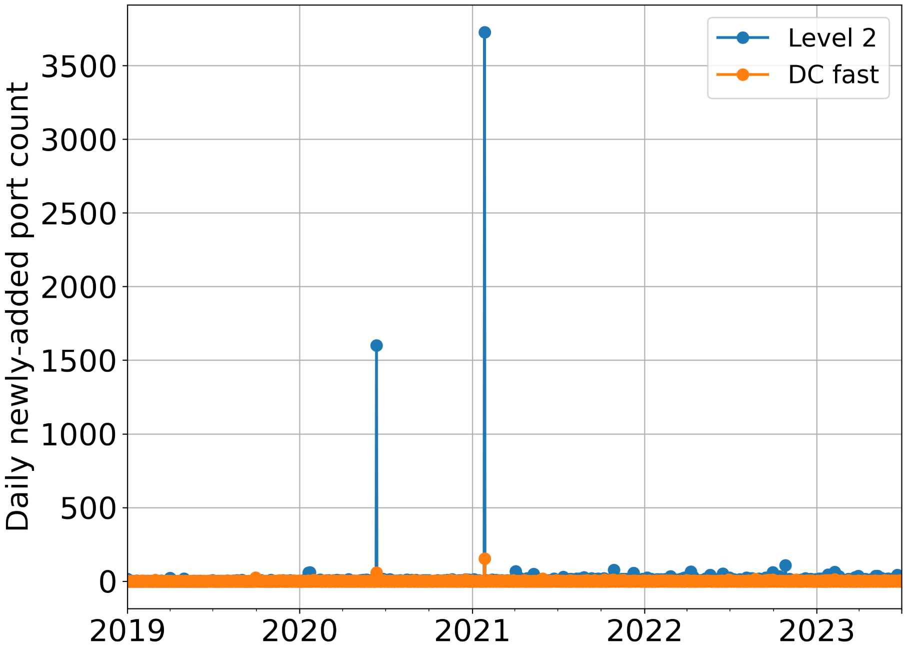

Figure S2: **Anomalous Surge in EV Charging Port Counts.** Anomalous surge of EV charging port counts due to integration of data from EV charger providers into the data center on June 12, 2020, and January 27, 2021 [20]. Data source: the United States Department of Energy's Alternative Fuels Data Center [19].

S3 Propensity score matching results

In this section, we present the results of the propensity score matching analysis. Table S3 displays the logistic regression results, identifying the determinants of EV charger deployment. Figures S3 and S4 illustrate the covariate balance after the matching process for the years 2019 and 2021-2023, respectively.

Table S3: Determinants of EV Charger Deployments

	Dependent variable: Within 500m of a EV charger	
	2019 Sample	2021-2023 Sample
	(1)	(2)
log (Population density)	0.071***	−0.018
	(0.024)	(0.025)
	p = 0.004	p = 0.466
log (Building density)	−0.129***	0.099***
	(0.020)	(0.009)
	p < 0.001	p < 0.001
log (Auto-oriented road miles per square mile)	0.112*	0.158***
	(0.061)	(0.026)
	p = 0.067	p < 0.001
log (Walkability Index score)	1.689***	1.819***
	(0.036)	(0.082)
	p < 0.001	p < 0.001
log (Median household income)	−0.028	0.620***
	(0.038)	(0.071)
	p = 0.452	p < 0.001
Employed population percentage	0.003	0.005***
	(0.004)	(0.001)
	p = 0.394	p < 0.001
Female percentage (%)	−0.048***	−0.001
	(0.006)	(0.002)
	p < 0.001	p = 0.494
White population (%)	−0.003	−0.002
	(0.003)	(0.002)
	p = 0.352	p = 0.254
Black population (%)	0.012***	0.0001
	(0.001)	(0.003)
	p < 0.001	p = 0.984
Native American population (%)	−0.127***	−0.019***
	(0.019)	(0.007)
	p < 0.001	p = 0.007
EV sales per 1000 people at the county level	0.304	−1.928***
	(0.437)	(0.035)
	p = 0.487	p < 0.001
log (Spending), without EV chargers	0.167***	0.212***
	(0.030)	(0.011)
	p < 0.001	p < 0.001
Constant	−6.053***	−14.140***
	(0.470)	(0.905)
	p < 0.001	p < 0.001
Observations	114,277	131,790
Log Likelihood	−23,009.370	−61,657.350
Akaike Inf. Crit.	46,044.740	123,340.700
Accuracy	0.946	0.796

Note: Standard errors, clustered by category of POI, are reported in parentheses, and p-values from two-sided t-tests are listed below the standard errors. Auto-oriented intersections per square mile is omitted because it highly correlates with Auto-oriented road miles per square mile. To avoid errors when taking the logarithm of zero, a value of one is added to all variables that are logged. *p<0.1; **p<0.05; ***p<0.01

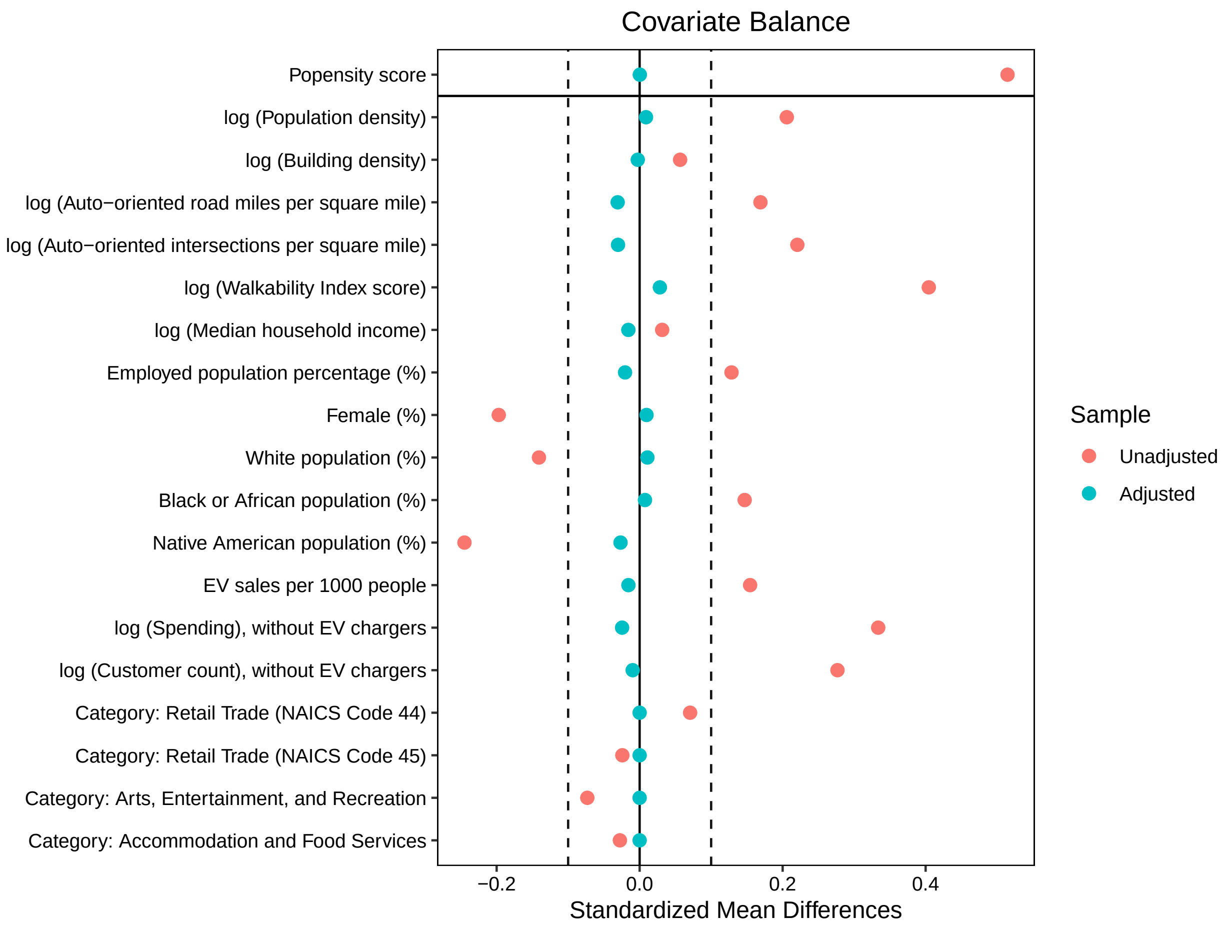

Figure S3: **Covariate balance (2019 sample).** $n = 114{,}277$ samples.

134 S4 Variation in treatment effects by distance

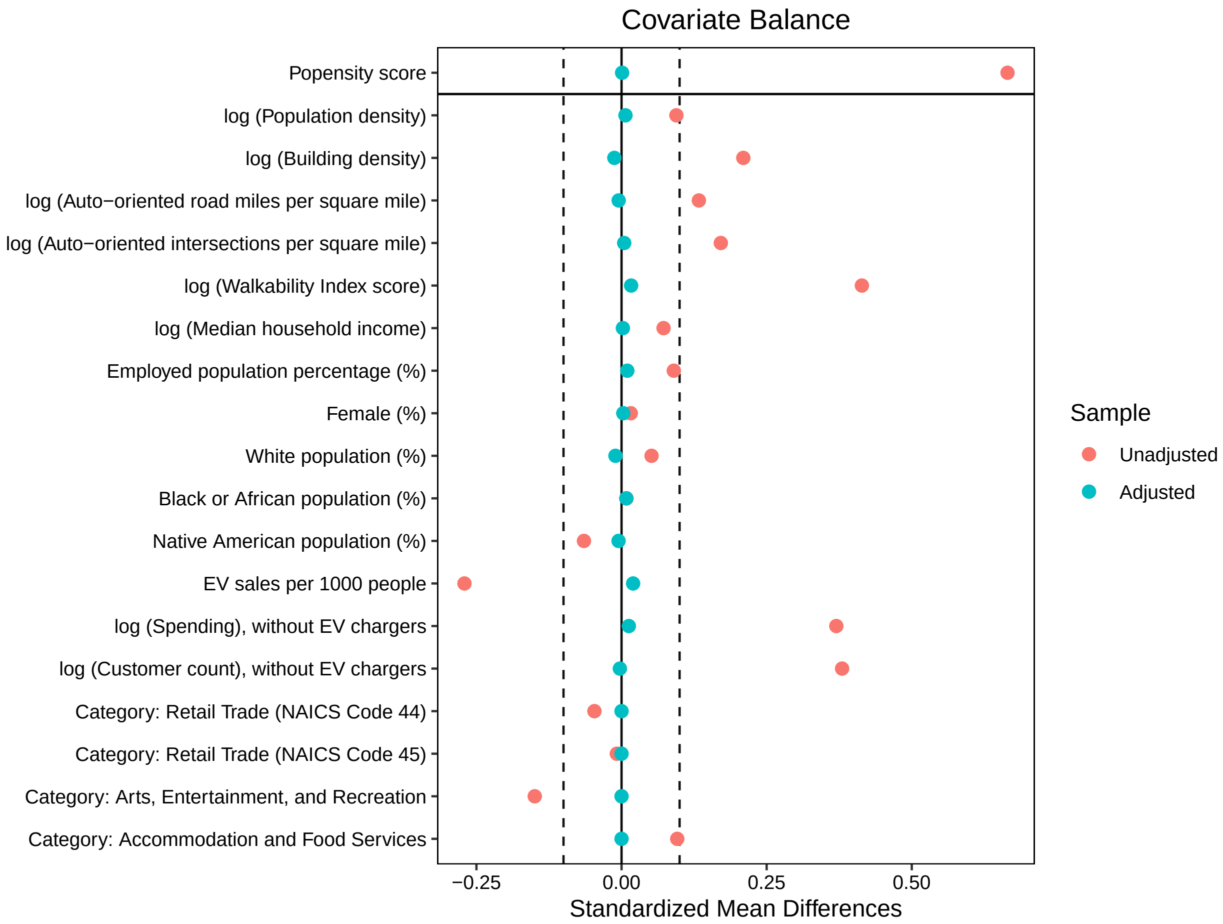

Figure S4: **Covariate balance (2021-2023 sample).** n = 131,790 samples.

Table S4: Variation in treatment effects by distance

	2019 Sample		2021-2023 Sample	
	Customer Count	Spending	Customer Count	Spending
	(1)	(2)	(3)	(4)
Treatment effects in:				
0 - 100m	0.0049**	0.0046***	0.0064***	0.0060***
	(0.0021)	(0.0014)	(0.0018)	(0.0017)
	p=0.0218	p=0.00136	p<0.001	p<0.001
100 - 200m	0.0022**	0.0020***	0.0019**	0.0017***
	(0.0010)	(0.0008)	(0.0008)	(0.0006)
	p=0.0289	p=0.00888	p=0.0224	p=0.00869
200 - 300m	0.0040***	0.0032***	0.0001	1.9×10^{-5}
	(0.0011)	(0.0009)	(0.0006)	(0.0005)
	p<0.001	p<0.001	p=0.854	p=0.972
300 - 400m	0.0010	0.0013*	0.0020***	0.0017***
	(0.0010)	(0.0007)	(0.0005)	(0.0004)
	p=0.341	p=0.0917	p<0.001	p<0.001
400 - 500m	0.0026**	0.0018**	0.0016***	0.0017***
	(0.0011)	(0.0009)	(0.0006)	(0.0005)
	p=0.0191	p=0.0445	p=0.00639	p<0.001
Fixed-effects				
Individual POI	Yes	Yes	Yes	Yes
County-by-month	Yes	Yes	Yes	Yes
Fit statistics				
Observations	133,649	133,649	1,235,819	1,235,819
R^2	0.93580	0.96236	0.86250	0.89915

Note: Clustered (placekey) standard-errors reported in parentheses, and p-values from two-sided t-tests are listed under standard errors. *p<0.1; **p<0.05; ***p<0.01.

S5 Heterogeneity of Effects by POI and EV Charger Type

In this section, we explore the heterogeneity of the effects by POI and EV charger type. Tables S5 and S6 provide estimates of the heterogeneous EVCS effects for customers' median distance from home and customers' median dwell time, respectively.

Table S5: Effects on median distance from home by EVCS types and POI types

Dependent Variable:	Median distance from home				
	All	Restaurant	Grocery/clothing store	Hotel	Gasoline Stations with Convenience Stores
	(1)	(2)	(3)	(4)	(5)
2019 Sample:					
Treatment effect (L1/L2 chargers)	0.0041**	0.0061**	0.0032	-0.0069	-0.0128
	(0.0017)	(0.0026)	(0.0044)	(0.0080)	(0.0135)
	p=0.0174	p=0.0203	p=0.464	p=0.385	p=0.343
Treatment effect (DC fast chargers)	0.0012	0.0014	0.0014	0.0114	0.0046
	(0.0013)	(0.0024)	(0.0025)	(0.0091)	(0.0054)
	p=0.349	p=0.556	p=0.583	p=0.208	p=0.394
Observations	133,649	58,768	25,361	3,557	3,193
R^2	0.90651	0.92556	0.86849	0.91203	0.85752
2021-2023 Sample:					
Treatment effect (L1/L2 chargers)	-0.0069***	-0.0048***	-0.0159***	-0.0035	-0.0010
	(0.0009)	(0.0009)	(0.0032)	(0.0029)	(0.0051)
	p<0.001	p<0.001	p<0.001	p=0.238	p=0.842
Treatment effect (DC fast chargers)	-0.0088***	-0.0052***	-0.0132***	-0.0029	-0.0193***
	(0.0009)	(0.0014)	(0.0021)	(0.0036)	(0.0062)
	p<0.001	p<0.001	p<0.001	p=0.419	p=0.00202
Observations	1,235,819	625,211	187,609	30,531	39,877
R^2	0.74681	0.74295	0.73940	0.80795	0.78767

*Note:*Clustered (placekey) standard-errors in parentheses, and p-values from two-sided t-tests are listed under standard errors. We controlled for individual POI, time, and county-by-time fixed effects. *p<0.1; **p<0.05; ***p<0.01.

Table S6: Effects on median dwell time by EVCS types and POI types

Dependent Variable:	Median dwell time				
	All	Restaurant	Grocery/clothing store	Hotel	Gasoline Stations with Convenience Stores
	(1)	(2)	(3)	(4)	(5)
2019 Sample:					
Treatment effect (L1/L2 chargers)	0.0016	0.0028	-0.0008	-0.0014	-0.0042
	(0.0014)	(0.0023)	(0.0030)	(0.0065)	(0.0059)
	p=0.239	p=0.212	p=0.801	p=0.824	p=0.474
Treatment effect (DC fast chargers)	0.0005	0.0006	0.0004	0.0061	-0.0022
	(0.0008)	(0.0014)	(0.0019)	(0.0059)	(0.0028)
	p=0.496	p=0.675	p=0.826	p=0.301	p=0.423
Observations	133,649	58,768	25,361	3,557	3,193
R^2	0.83895	0.84079	0.80836	0.91732	0.83585
2021-2023 Sample:					
Treatment effect (L1/L2 chargers)	-0.0063***	-0.0058***	-0.0107***	-0.0100***	0.0044**
	(0.0006)	(0.0007)	(0.0019)	(0.0037)	(0.0017)
	p<0.001	p<0.001	p<0.001	p=0.00729	p=0.0125
Treatment effect (DC fast chargers)	-0.0020***	-0.0011	-0.0017	-0.0214***	0.0009
	(0.0007)	(0.0010)	(0.0013)	(0.0066)	(0.0013)
	p=0.00219	p=0.263	p=0.194	p=0.00122	p=0.496
Observations	1,235,819	625,211	187,609	30,531	39,877
R^2	0.66639	0.65070	0.61957	0.83461	0.71442

*Note:*Clustered (placekey) standard-errors in parentheses, and p-values from two-sided t-tests are listed under standard errors. We controlled for individual POI, time, and county-by-time fixed effects. *p<0.1; **p<0.05; ***p<0.01.

S6 Effects of EVCS on customer counts by different income groups

Table S7: Effects of EVCS on customer counts by different income groups

Customer count with income	<$25K	$25K-$45K	$45K-$65K	$60K-$75K	$75K-$100K	$100K-$150K	>$150K
	(1)	(2)	(3)	(4)	(5)	(6)	(7)
2019 Sample:							
Treatment effect	0.0018***	0.0012**	0.0014***	0.0022***	0.0010**	0.0022***	0.0015***
	(0.0005)	(0.0005)	(0.0005)	(0.0005)	(0.0005)	(0.0005)	(0.0005)
	p<0.001	p=0.0176	p=0.00353	p<0.001	p=0.0427	p<0.001	p=0.00171
Observations	133,649	133,649	133,649	133,649	133,649	133,649	133,649
R^2	0.89682	0.91276	0.89258	0.86722	0.89942	0.90937	0.92359
2021-2023 Sample:							
Treatment effect	0.0004*	0.0005**	0.0002	0.0003	0.0007***	0.0011***	0.0015***
	(0.0002)	(0.0002)	(0.0002)	(0.0002)	(0.0002)	(0.0002)	(0.0002)
	p=0.058	p=0.0223	p=0.329	p=0.124	p=0.00247	p<0.001	p<0.001
Observations	1,235,819	1,235,819	1,235,819	1,235,819	1,235,819	1,235,819	1,235,819
R^2	0.74790	0.77726	0.74797	0.71813	0.76314	0.78659	0.81846

*Note:*Clustered (placekey) standard-errors in parentheses, and p-values from two-sided t-tests are listed under standard errors. We controlled for individual POI, time, and county-by-time fixed effects. *p<0.1; **p<0.05; ***p<0.01.

S7 Sensitivity analysis regarding disadvantaged community definition

In this section, we explore the sensitivity of our modeling results to different definitions of disadvantaged communities. In our primary model (Table 1), we assess the impact of EVCS on local businesses within communities identified as disadvantaged or low-income by both California and Justice40 initiatives [21]. Here, we relax this criterion and examine the results when businesses are located within communities identified as disadvantaged communities designated by California and Justice40 separately. The estimation results of the treatment effect (Table S8) indicate that the impacts of EVCS installation on customer count and spending remain significant under these alternative criteria. Furthermore, the effect sizes closely mirror those observed in the primary model, demonstrating the robustness of our findings to variations in disadvantaged community definitions.

Table S8: Impacts of EVCSs under two different underprivileged community definitions.

	2019 Sample				2021-2023 Sample			
	Justice40		CA criteria		Justice40		CA criteria	
Dependent variables:	Customer Count	Spending	Customer Count	Spending	Customer Count	Spending	Customer Count	Spending
	(1)	(2)	(3)	(4)	(5)	(6)	(7)	(8)
Variables								
Treatment effect	0.0015**	0.0031***	0.0010*	0.0018**	0.0012***	0.0014***	0.0009***	0.0009**
	(0.0006)	(0.0008)	(0.0006)	(0.0008)	(0.0003)	(0.0003)	(0.0003)	(0.0004)
	p=0.0108	p<0.001	p=0.0773	p=0.027	p<0.001	p<0.001	p=0.00123	p=0.0104
Fixed-effects								
Individual POI	Yes	Yes	Yes	Yes	Yes	Yes	Yes	Yes
County-by-month	Yes	Yes	Yes	Yes	Yes	Yes	Yes	Yes
Fit statistics								
Observations	75,148	75,148	76,008	76,008	681,004	681,004	715,147	715,147
R^2	0.95993	0.93177	0.96071	0.93386	0.89635	0.85811	0.89644	0.85868

Note: Clustered (at the POI level) standard-errors in parentheses, and p-values from two-sided t-tests are listed below the standard errors. The dependent variables are the natural log of the number of customers and the natural log of total customer spending, respectively. "Justice40" represents disadvantaged communities designated by the Justice40 Initiative [22], and "CA criteria" stands for low-income or disadvantaged communities designated by California [23], *p<0.1; **p<0.05; ***p<0.01

S8 EVCS costs

Table S9 provides an approximate overview of electric vehicle charging station infrastructure costs in California. The hardware and installation costs[1] are derived from a whitepaper by the International Council on Clean Transportation (ICCT) [24], in which the authors calculate the numbers based on several research studies, including those by Ducharme & Kargas [25], the Rocky Mountain Institute [26], Avista Utility [27], DOE [28], ClipperCreek [29], ChargePoint [30], and others [31, 28, 32]. It's important to note that these costs can vary significantly based on factors such as the features of the EVSE unit, site location, available electrical capacity, and labor costs. However, we have cross-validated these estimates with other reports, including a 2019 report by the Rocky Mountain Institute [33] and a 2015 report by the DOE [34], and found general consistency in the order of magnitude.

To estimate the average total cost, we combine the simple average hardware cost with the average installation cost. The installation cost is weighted based on the percentage distribution

[1] Hardware costs for public and workplace charging infrastructure cover the charger and its pedestal. The primary factors affecting these costs are the power output of the unit, whether a pedestal is needed, and whether the unit is networked with communication or payment processing capabilities [24]. Our assumption is that Level 2 and DC fast chargers are both networked. Installation costs include expenses for labor, materials, permits, taxes, and utility upgrades [24].

of the number of chargers at each site. According to data from the United States Department of Energy's Alternative Fuels Data Center [19], for Level 2 charging stations between 2021 and 2023, 23% of sites have one charger, 37% have two chargers, 20% have three to five chargers, and 20% have more than five chargers. For DC fast chargers during the same period, 17% of sites have one charger, 16% have two chargers, 27% have three to five chargers, and 40% have more than five chargers. By incorporating this distribution pattern along with the cost data, we can calculate the estimated average total cost per charger for each charger type as shown in the last column of Table S9.

We then multiply the estimated average total cost by the average number of ports per charging station, which is 5.0 for 2021-2023. As a result, the estimated average cost for a charging station is approximately $30,790 for a Level 2 EVCS, $281,430 for a 50 kW DC fast chargers, $521,400 for a 150 kW DC fast chargers, and $902,170 for a 350 kW DC fast chargers.

Table S9: EVCS infrastructure costs per charger

**Charger Type**	**Hardware Cost**		**Installation Cost**				**Total Cost**
	No. of chargers/pedestal:		*No. of chargers per site:*				(Estimated Average)
	1	2	1	2	3-5	6+	
Level 1	$813	$596	N/A	N/A	N/A	N/A	$705
Level 2	$3,127	$2,793	$4,148	$3,039	$2,745	$2,837	$6,158
DC Fast (50 kW)	$28,401	N/A	$45,506	$36,235	$26,964	$17,692	$56,286
DC Fast (150 kW)	$75,000	N/A	$47,781	$38,047	$28,312	$18,577	$104,280
DC Fast (350 kW)	$140,000	N/A	$65,984	$52,541	$39,097	$25,654	$180,434

References

[1] U.S. Department of Transportation, Charger types and speeds, `https://www.transportation.gov/rural/ev/toolkit/ev-basics/charging-speeds`, last accessed on 2024-03-18 (2023).

[2] US Department of Energy's Alternative Fuels Data Center, Charging electric vehicles at home, `https://afdc.energy.gov/fuels/electricity_charging_home.html`, last accessed on 2024-03-18.

[3] S. LaMonaca, L. Ryan, The state of play in electric vehicle charging services–a review of infrastructure provision, players, and policies, Renewable and sustainable energy reviews 154 (2022) 111733.

[4] US Department of Energy's Alternative Fuels Data Center, Charging electric vehicles in public, `https://afdc.energy.gov/fuels/electricity_charging_public.html`, last accessed on 2024-03-18.

[5] J. H. Lee, S. J. Hardman, G. Tal, Who is buying electric vehicles in california? characterising early adopter heterogeneity and forecasting market diffusion, Energy Research & Social Science 55 (2019) 218–226.

[6] E. Muehlegger, D. Rapson, Understanding the distributional impacts of vehicle policy: who buys new and used alternative vehicles?, National Center for Sustainable Transportation (2018).

[7] Z. Farkas, H.-S. Shin, A. Nickkar, Environmental attributes of electric vehicle ownership and commuting behavior in maryland: Public policy and equity considerations, Mid-Atlantic Transportation Sustainability University Transportation Center. Retrieved April 20 (2018) 2019.

[8] J. H. Lee, D. Chakraborty, S. J. Hardman, G. Tal, Exploring electric vehicle charging patterns: Mixed usage of charging infrastructure, Transportation Research Part D: Transport and Environment 79 (2020) 102249.

[9] G. Tal, S. S. Raghavan, V. C. Karanam, M. P. Favetti, K. M. Sutton, J. Lee, C. Nitta, D. Chakraborty, M. Nicholas, T. Turrentine, Advanced plug-in electric vehicle travel and charging behavior final report, California Air Resources Board Contract (2020) 12–319.

[10] A. Almaghrebi, S. Shom, F. Al Juheshi, K. James, M. Alahmad, Analysis of user charging behavior at public charging stations, in: 2019 IEEE Transportation Electrification Conference and Expo (ITEC), IEEE, 2019, pp. 1–6.

[11] P. Morrissey, P. Weldon, M. O'Mahony, Future standard and fast charging infrastructure planning: An analysis of electric vehicle charging behaviour, Energy policy 89 (2016) 257–270.

[12] B. Borlaug, F. Yang, E. Pritchard, E. Wood, J. Gonder, Public electric vehicle charging station utilization in the united states, Transportation Research Part D: Transport and Environment 114 (2023) 103564.

[13] D. Garas, G. O. Collantes, M. A. Nicholas, City of vancouver ev infrastructure strategy report (2016).

[14] California Air Resources Board, Proposed fiscal year 2023-24 funding plan for clean transportation incentives for low carbon transportation investments and the air quality improvement program., `https://ww2.arb.ca.gov/sites/default/files/2023-10/Proposed%20Funding%20Plan%20Fiscal%20Year%202023-24.pdf`, last accessed on 2024-03-21 (2024).

[15] T. House, Fact sheet: Biden-harris administration proposes new standards for national electric vehicle charging network (2022).

[16] Center for Sustainable Energy, Rebate statistics dashboard, `https://calevip.org/rebate-statistics`, last accessed on 2024-03-21 (2024).

[17] OEHHA, Sb 535 disadvantaged communities, https://oehha.ca.gov/calenviroscreen/sb535#:~:text=Senate%20Bill%20535%20(De%20Le%C3%B3n,projects%20located%20within%20those%20communities, last accessed on 2024-03-21 (2024).

[18] C.-W. Hsu, K. Fingerman, Public electric vehicle charger access disparities across race and income in california, Transport Policy 100 (2021) 59–67.

[19] US Department of Energy's Alternative Fuels Data Center, Electric vehicle charging station locations, https://afdc.energy.gov/fuels/electricity_locations.html#/find/nearest?fuel=ELEC (2023).

[20] A. Brown, A. Schayowitz, E. Klotz, Electric vehicle charging infrastructure trends from the alternative fueling station locator: First quarter 2021, Tech. rep., National Renewable Energy Laboratory (NREL) (2021).

[21] California Energy Commission, California and justice40 disadvantaged or low-income communities, https://cecgis-caenergy.opendata.arcgis.com/datasets/ec0adaef7db349dfa584ee33ea4c3f1f_0/explore, last accessed on 2024-03-21 (2024).

[22] California Energy Commission, Disadvantaged communities designated by justice40, https://cecgis-caenergy.opendata.arcgis.com/datasets/6aae26a55d834ba18de6844e14371334_0/explore, last accessed on 2024-03-21 (2024).

[23] California Energy Commission, Low-income or disadvantaged communities designated by california, https://cecgis-caenergy.opendata.arcgis.com/datasets/4c148fc005b3453faa645a97c72b6f21_0/explore?location=36.957353%2C-119.179789%2C6.22, last accessed on 2024-03-21 (2024).

[24] M. Nicholas, Estimating electric vehicle charging infrastructure costs across major us metropolitan areas, ICCT Washington, DC, USA, 2019.

[25] P. Ducharme, C. Kargas, Feasibility of a pan-canadian network of dc fast charging stations for evs, World Electric Vehicle Journal 8 (1) (2016) 1–13.

[26] J. Agenbroad, B. Holland, Pulling back the veil on ev charging station costs, Rocky Mountain Institute blog 29 (2014).

[27] A. Utility, Docket no. ue-160082 – avista utilities semi-annual report on electric vehicle supply equipment pilot program, https://www.utc.wa.gov/_layouts/15/CasesPublicWebsite/CaseItem.aspx?item=document&id=00044&year=2016&docketNumber=160082&resultSource=&page=&query=&refiners=&isModal=&omItem=false&doItem=false, last accessed on 2024-03-08 (2018).

[28] M. W. Melaina, G. Heath, D. Sandor, D. Steward, L. Vimmerstedt, E. Warner, K. W. Webster, Transportation energy futures series: Alternative fuel infrastructure expansion: Costs,

resources, production capacity, and retail availability for low-carbon scenarios, Tech. rep., National Renewable Energy Lab.(NREL), Golden, CO (United States) (2013).

[29] ClipperCreek, Commercial evse: Public, fleet, workplace charging, https://store.clippercreek.com/commercial, last accessed on 2019-02-01 (2019).

[30] Chargepoint, Smart charging stations, https://www.chargepoint.com/products/commercial/, last accessed on 2019-02-01.

[31] J. DeShazo, N. Wong, J. Karpman, Overcoming barriers to electric vehicle charging in multi-unit dwellings: A westside cities case study (2017).

[32] H. Ribberink, L. Wilkens, R. Abdullah, M. McGrath, M. Wojdan, Impact of clusters of dc fast charging stations on the electricity distribution grid in ottawa, canada, in: Proceedings of the Electric Vehicle Symposium, Vol. 30, 2017.

[33] C. Nelder, E. Rogers, Reducing ev charging infrastructure costs, Rocky Mountain Institute 17 (2019).

[34] U. D. of Energy, Costs associated with non-residential electric vehicle supply equipment: Factors to consider in the implementation of electric vehicle charging stations, Tech. rep. (2015).
