# Supplementary material for: Effects of electric vehicle charging stations on the economic vitality of local businesses
Source: Nat Commun. 2024 Sep 4;15:7437. doi: 10.1038/s41467-024-51554-9 (PMC11375121; doi:10.1038/s41467-024-51554-9)
Supplement: Supplementary file 3 — Reporting Summary [file 41467_2024_51554_MOESM3_ESM.pdf]

nature portfolio

Corresponding author(s): Yunhan Zheng

Last updated by author(s): Jul 18, 2024

Reporting Summary

Nature Portfolio wishes to improve the reproducibility of the work that we publish. This form provides structure for consistency and transparency in reporting. For further information on Nature Portfolio policies, see our Editorial Policies and the Editorial Policy Checklist.

Statistics

For all statistical analyses, confirm that the following items are present in the figure legend, table legend, main text, or Methods section.

n/a	Confirmed	
☐	☒	The exact sample size (*n*) for each experimental group/condition, given as a discrete number and unit of measurement
☐	☒	A statement on whether measurements were taken from distinct samples or whether the same sample was measured repeatedly
☐	☒	The statistical test(s) used AND whether they are one- or two-sided<br>*Only common tests should be described solely by name; describe more complex techniques in the Methods section.*
☐	☒	A description of all covariates tested
☐	☒	A description of any assumptions or corrections, such as tests of normality and adjustment for multiple comparisons
☐	☒	A full description of the statistical parameters including central tendency (e.g. means) or other basic estimates (e.g. regression coefficient) AND variation (e.g. standard deviation) or associated estimates of uncertainty (e.g. confidence intervals)
☐	☒	For null hypothesis testing, the test statistic (e.g. *F*, *t*, *r*) with confidence intervals, effect sizes, degrees of freedom and *P* value noted<br>*Give P values as exact values whenever suitable.*
☒	☐	For Bayesian analysis, information on the choice of priors and Markov chain Monte Carlo settings
☒	☐	For hierarchical and complex designs, identification of the appropriate level for tests and full reporting of outcomes
☒	☐	Estimates of effect sizes (e.g. Cohen's *d*, Pearson's *r*), indicating how they were calculated

Our web collection on statistics for biologists contains articles on many of the points above.

Software and code

Policy information about availability of computer code

Data collection	No specific software is used for data collection.
Data analysis	Data processing utilized Python version 3.7.4 and R version 3.6.3, while modeling was performed using R version 3.6.3. The code used for conducting the analysis is accessible on GitHub at https://github.com/zhengyunhan/EVCS_economic_vitality.

For manuscripts utilizing custom algorithms or software that are central to the research but not yet described in published literature, software must be made available to editors and reviewers. We strongly encourage code deposition in a community repository (e.g. GitHub). See the Nature Portfolio guidelines for submitting code & software for further information.

Data

Policy information about availability of data

All manuscripts must include a data availability statement. This statement should provide the following information, where applicable:

- Accession codes, unique identifiers, or web links for publicly available datasets
- A description of any restrictions on data availability
- For clinical datasets or third party data, please ensure that the statement adheres to our policy

The Point-of-Interest-related data utilized in this study is not publicly available but can be requested directly from Dewey (https://www.deweydata.io/). All other data have been sourced from publicly available channels, and the specific sources for each variable are detailed in the Data section within the Methods.

Research involving human participants, their data, or biological material

Policy information about studies with human participants or human data. See also policy information about sex, gender (identity/presentation), and sexual orientation and race, ethnicity and racism.

Reporting on sex and gender	n/a
Reporting on race, ethnicity, or other socially relevant groupings	n/a
Population characteristics	n/a
Recruitment	n/a
Ethics oversight	n/a

Note that full information on the approval of the study protocol must also be provided in the manuscript.

Field-specific reporting

Please select the one below that is the best fit for your research. If you are not sure, read the appropriate sections before making your selection.

☐ Life sciences ☒ Behavioural & social sciences ☐ Ecological, evolutionary & environmental sciences

For a reference copy of the document with all sections, see nature.com/documents/nr-reporting-summary-flat.pdf

Behavioural & social sciences study design

All studies must disclose on these points even when the disclosure is negative.

Study description	Quantitative panel data analysis. We identify the impacts of electric vehicle charging stations installations on customer counts and spending at surrounding Points-of-Interest (POI) in California during 2019 as well as between January 2021 and June 2023, employing a Difference-in-Differences approach.
Research sample	We gather data pertaining to California in 2019 and between January 2021 and June 2023, including information on (1) electric vehicle charging stations opening during either of these two periods, detailing their locations and open dates; (2) Point-of-Interest (POI) data, specifying locations and attributes such as categories; (3) monthly customer counts, spending, median distance from home, median dwell time, and customer income distribution data at these POIs; and (4) socio-demographic data at the census tract level. The research sample consists of POIs in California, sourced from Safegraph and Advan. These sources cover a wide range of POI categories and provide up-to-date records of locations and detailed attributes for each place, making them representative and capable of illustrating local business dynamics. Please note that the POI-related data utilized in this study is not publicly available but can be requested from Dewey (https://www.deweydata.io/). All other data were sourced from publicly available channels.
Sampling strategy	Information about electric vehicle charging stations is sourced from the United States Department of Energy's Alternative Fuels Data Center. Point-of-Interest (POI) data is acquired from Dewey, a collaborative effort with Safegraph and Advan. The sampling strategy employed ensures comprehensive representation by collecting data from a diverse set of users and devices, a validity that is further confirmed through benchmarking.
Data collection	Data collection was done by he United States Department of Energy's Alternative Fuels Data Center and Dewey.
Timing	Our data analysis spans two distinct time periods: the entirety of 2019 and from January 2021 to June 2023
Data exclusions	No data was excluded.
Non-participation	N/A
Randomization	N/A

Reporting for specific materials, systems and methods

We require information from authors about some types of materials, experimental systems and methods used in many studies. Here, indicate whether each material, system or method listed is relevant to your study. If you are not sure if a list item applies to your research, read the appropriate section before selecting a response.

Materials & experimental systems

n/a	Involved in the study
☒	☐ Antibodies
☒	☐ Eukaryotic cell lines
☒	☐ Palaeontology and archaeology
☒	☐ Animals and other organisms
☒	☐ Clinical data
☒	☐ Dual use research of concern
☒	☐ Plants

Methods

n/a	Involved in the study
☒	☐ ChIP-seq
☒	☐ Flow cytometry
☒	☐ MRI-based neuroimaging

Plants

Seed stocks	N/A
Novel plant genotypes	N/A
Authentication	N/A
